# Supplementary material for: Drug repurposing for aging research using model organisms
Source: Aging Cell. 2017 Jun 16;16(5):1006–15. doi: 10.1111/acel.12626 (PMC5595691; doi:10.1111/acel.12626)
Supplement: Supplementary file 7 — Data S1 Zip‐Archive of all report cards. [file ACEL-16-1006-s007.zip › RC_KRG.pdf]

KRG

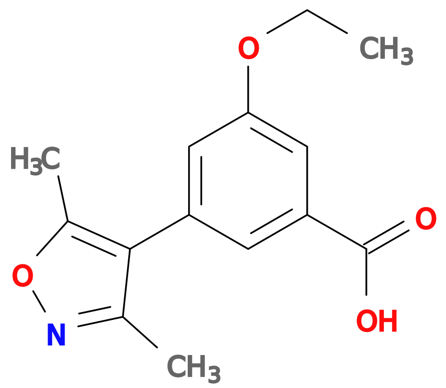

Database identifiers
ChEMBLCompound CHEMBL1828984

Ranking

	Rank	Score
Drosophila	688/697	0.025
C. elegans	582/591	0.0

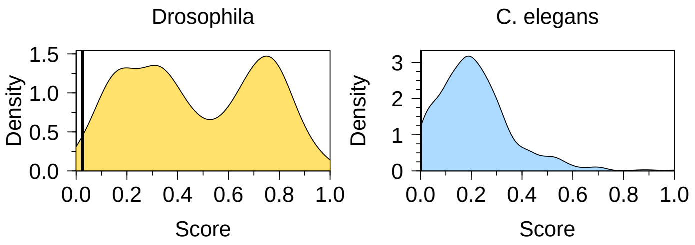

	Ageing implication	Domain conservation	Binding site conservation	Binding affinity	Bioavailability	Lipinski	Promiscuity	Purchasability	Drug approval	**Total**
Drosophila	0.203	0.625	0.729	0.307	(0.9)	0.0	-0.0	0.0	0.0	0.025
C. elegans	0.203	0.005	0.0	0.307	0.273	0.0	-0.0	0.0	0.0	0.0

Names

No synonyms found

Roles

ChEBI entry None has no roles

Status

Approved drug (according to ChEMBL)	No
Number of Rule of 5 violations	0
Binding affinity to original target in log units (RF-Score prediction)	4.18
Burns *C. elegans* bioavailability prediction	-1.16

Compound Target Characteristics

CREB-binding protein

Best gene implication in ageing for this target family came from gene Q92793 via mapping the annotation from Ensembl ENSG00000005339 via mapping the annotation from EntrezGene 1387 via mapping the annotation from GenAgeHuman 0064 annotated in GenAge release 17.

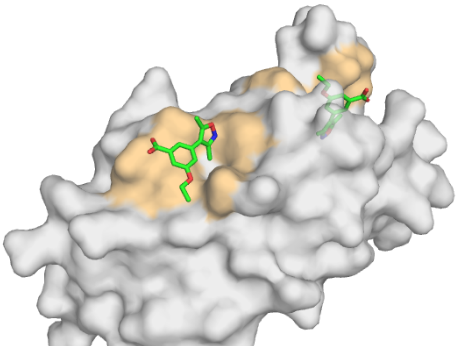

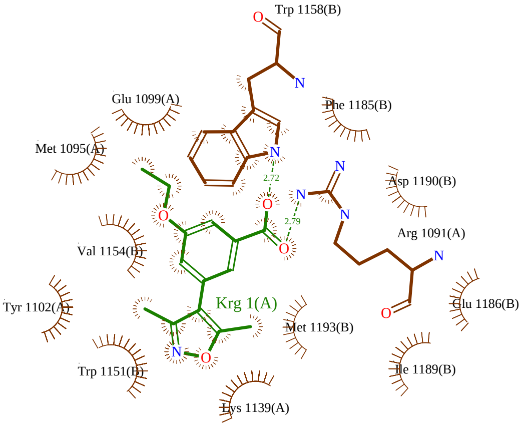

protein	amino acids contacts (binding site)													
PDB:3svh:chainA:Q92793	R	M	E	Y	P	F	Q	V	L	I	Y	K	N	V
sp:Q09472:EP300_HUMAN	R	M	E	Y	P	F	Q	V	L	I	Y	K	N	V
sp:Q92793:CBP_HUMAN	R	M	E	Y	P	F	Q	V	L	I	Y	K	N	V
tr:F1M9G7:F1M9G7_RAT	R	M	E	Y	P	F	Q	V	L	I	Y	K	N	V
tr:Q8QZV8:Q8QZV8_MOUSE	R	M	E	Y	P	F	Q	V	L	I	Y	K	N	V
tr:F8VPR5:F8VPR5_MOUSE	R	M	E	Y	P	F	Q	V	L	I	Y	K	N	V
tr:Q8T3Z6:Q8T3Z6_DROME	L	K	N	W	H	F	H	V	L	V	Y	R	N	V
tr:Q058W2:Q058W2_DROME	L	K	N	W	H	F	H	V	L	V	Y	R	N	V
tr:Q9VCG6:Q9VCG6_DROME	L	K	N	W	H	F	H	V	L	V	Y	R	N	V
tr:Q8T3Z8:Q8T3Z8_DROME	L	K	L	A	D	F	E	V	L	V	Y	V	N	V
tr:Q4V5A7:Q4V5A7_DROME	L	K	L	A	D	F	E	V	L	V	Y	V	N	V
tr:O17681:O17681_CAEEL	-	-	-	-	-	-	-	-	-	-	-	-	-	-
tr:Q9TZB8:Q9TZB8_CAEEL	-	-	-	-	-	-	-	-	-	-	-	-	-	-
sp:Q07442:BDF2_YEAST	-	K	S	N	P	F	Q	V	L	L	Y	S	N	V
sp:P35817:BDF1_YEAST	-	K	A	H	P	F	E	V	M	L	Y	A	N	V

	whole protein		domain-based		contact-based	
protein	ident	simil	ident	simil	ident	simil
PDB:3svh:chainA:Q92793	1.0	1.0	1.0	1.0	1.0	1.0
sp:Q09472:EP300_HUMAN	0.59	0.81	0.86	0.96	1.0	1.0
sp:Q92793:CBP_HUMAN	1.0	1.0	1.0	1.0	1.0	1.0
tr:F1M9G7:F1M9G7_RAT	0.96	0.99	0.98	0.99	1.0	1.0
tr:Q8QZV8:Q8QZV8_MOUSE	0.95	0.98	0.98	1.0	1.0	1.0
tr:F8VPR5:F8VPR5_MOUSE	0.96	0.99	0.98	1.0	1.0	1.0
tr:Q8T3Z6:Q8T3Z6_DROME	0.02	0.05	0.31	0.74	0.43	0.73
tr:Q058W2:Q058W2_DROME	0.02	0.09	0.3	0.74	0.43	0.73
tr:Q9VCG6:Q9VCG6_DROME	0.03	0.12	0.31	0.74	0.43	0.73
tr:Q8T3Z8:Q8T3Z8_DROME	0.02	0.14	0.19	0.65	0.43	0.55
tr:Q4V5A7:Q4V5A7_DROME	0.02	0.14	0.19	0.65	0.43	0.55
tr:O17681:O17681_CAEEL	0.01	0.04	0.0	0.0	0.0	0.0
tr:Q9TZB8:Q9TZB8_CAEEL	0.03	0.07	0.0	0.0	0.0	0.0
sp:Q07442:BDF2_YEAST	0.03	0.15	0.29	0.6	0.57	0.56
sp:P35817:BDF1_YEAST	0.03	0.16	0.27	0.61	0.43	0.57

tbrd-1 (FBgn0039124) associated phenotypes

male fertile

(Information from FlyBase)
